# Screening of Metabolite Biomarkers for Antioxidant and Glycosidase Inhibitory Activities from Oat Resources

**DOI:** 10.3390/foods15203740

**Published:** 2026-10-20

**Authors:** Haitao Zhou, Huiyong Yang, Yajie Zhang, Rui Hou, Yingying Zhu, Liang Zou

**Affiliations:** 1 College of Food and Bioengineering, Zhengzhou University of Light Industry, Zhengzhou 450001, China; 2 Zhangjiakou Academy of Agricultural Sciences, Zhangjiakou 075000, China; 3 College of Agronomy, Hebei Agricultural University, Baoding 071001, China; 4 Key Laboratory of Coarse Cereal Processing of Ministry of Agriculture and Rural Affairs, Chengdu University, Chengdu 610106, China; 5 Chengdu Agricultural College, Chengdu 611130, China

**Keywords:** oat, DPPH scavenging activity, α-Glucosidase inhibition, metabolic biomarker

## Abstract

Oat accessions vary substantially in their bioactive components and associated functional properties, but the metabolites underlying differences in the bioactivities of digested oat samples remain insufficiently characterized. In this study, samples obtained after simulated gastrointestinal digestion of 43 oat accessions were evaluated for 2,2-diphenyl-1-picrylhydrazyl (DPPH) radical-scavenging and α-glucosidase inhibitory activities, and their metabolite profiles were characterized using ultra-performance liquid chromatography–electrospray ionization–tandem mass spectrometry. Eleven differential metabolites were detected between the groups with high and low DPPH radical-scavenging activity, while nine were detected between groups with contrasting α-glucosidase inhibitory activities. Random forest-based feature selection identified tricin 7-O-β-D-glucoside, myricetin 3-O-glucoside, quercetin 3-*O*-galactoside, and β-sitosterol as candidate markers associated with variation in DPPH radical-scavenging activity. The association of β-sitosterol with the high-DPPH phenotype was interpreted as statistical rather than as evidence of direct radical-scavenging activity. Myricetin 3-*O*-glucoside, quercetin 3-*O*-galactoside, and reduced glutathione were identified as metabolic markers associated with α-glucosidase inhibition. The results revealed substantial variation in the in vitro functional properties and metabolite profiles of digested oat samples. These metabolic markers provide a new research basis for the efficient development and utilization of functional products.

## 1. Introduction

Oat (*Avena sativa* L.), a member of the Poaceae family, is the sixth most widely cultivated cereal crop globally, with a production volume surpassed only by maize, wheat, rice, barley, and sorghum. Its cultivation is primarily confined to temperate regions, specifically within the 40° to 60° latitude zones [1]. Hull-less oat, domesticated from the wild species *Avena fatua* L., has a cultivation history in China spanning over two millennia. It accounts for roughly 90% of the oat accessions grown domestically, constituting a substantial germplasm resource. This national collection encompasses approximately 1500 hull-less oats [2]. In China, oat cultivation is primarily concentrated in the northern regions, with major production bases in Inner Mongolia, Hebei, Shanxi, Gansu, Ningxia, and Xinjiang [3]. These regionally adapted cultivars exhibit strong environmental resilience and confer significant economic value. Oats are nutritionally dense, containing essential nutrients such as minerals, lipids, proteins, vitamins, and dietary fiber, in addition to unsaturated fatty acids and a diverse array of phytochemicals. These bioactive compounds are efficacious in mitigating the risk of various chronic diseases, including cardiovascular disease, colorectal cancer, and type 2 diabetes [4].

Oats have gained increasing global recognition for their high nutritional value and health benefits, particularly in relation to obesity and diabetes prevention and management. However, oats from diverse geographical and genetic backgrounds exhibit substantial variation in their agronomic, processing, and functional properties. For example, a comparative study of five oat accessions cultivated under different environmental conditions revealed significant inter-varietal differences in phenolic content, fatty acid composition, and antioxidant capacity [5]. Brindzová et al. [6] demonstrated that black oat accessions possess significantly higher total phenolic content and greater antioxidant capacity than yellow or hull-less accessions. Avenanthramides, a class of phenolic alkaloids unique to oats, demonstrate potent antioxidant, anti-inflammatory, and anti-allergic activities. Their concentration varies substantially among oat accessions, with Avenanthramide C (AVN-C) exhibiting the most potent antioxidant capacity [7]. A comparative analysis of 16 Chinese oat accessions by Ma et al. [8] revealed significant varietal differences in fatty acid composition, triacylglycerol profile, and lipid-associated compounds. The observed antioxidant capacity was positively correlated with the concentrations of polyphenols, squalene, and campesterol. The considerable variation in overall oat quality presents a major challenge for the sustainable development of the oat industry. Consequently, a key objective in modern oat breeding is to move beyond conventional targets, such as high yield and disease resistance, toward functional breeding strategies that prioritize health-promoting properties and quality stability.

The success of functional oat breeding relies on the precise identification of bioactive compounds responsible for their documented health benefits. Some digestion-derived metabolites may subsequently become available for intestinal absorption and contribute to physiological effects. For example, during simulated gastrointestinal digestion, oat polyphenols, including ferulic acid, other phenolic acids, and avenanthramides (AVNs), can be released from their bound forms. This release into free forms results in a marked increase in antioxidant capacity [9]. During gastrointestinal digestion, oats release or undergo transformation of various bioactive constituents, including bioactive peptides and polyphenol-derived compounds. In contrast, short-chain fatty acids (SCFAs), such as acetate, propionate, and butyrate, are generated primarily through microbial fermentation of indigestible oat components, particularly dietary fiber, in the colon [10]. These compounds act synergistically to inhibit key metabolic enzymes, such as dipeptidyl peptidase-IV (DPP-IV), angiotensin-converting enzyme (ACE), and α-glucosidase, while promoting the proliferation of beneficial gut bacteria and stimulating the production of propionate and acetate. Through these multi-faceted mechanisms, they collectively contribute to reductions in blood glucose, blood pressure, and cholesterol levels, as well as the modulation of gut microbiota composition [11]. Therefore, a comprehensive analysis of post-digestion metabolite profiles across diverse oat germplasm, combined with an assessment of inter-varietal differences and the identification of key bioactive metabolites, will establish a critical scientific foundation for functional oat breeding programs.

In recent years, ultra-performance liquid chromatography-tandem mass spectrometry (UPLC-MS/MS) has been extensively employed in metabolomics for the comprehensive profiling of plant metabolites, owing to its high throughput, rapid separation, high sensitivity, and broad coverage [12]. The identification and analysis of differential metabolites facilitate the discovery of metabolic biomarkers, providing a modern paradigm for the breeding of novel crop accessions. For instance, Wang et al. [13] identified kaempferol-3-*O*-hexoside, kaempferol-7-*O*-glucoside, and naringenin-*O*-hexoside as key biomarkers in Tartary buckwheat associated with high flavonoid content and antioxidant activity. Xiong et al. [14] identified tricin and schaftoside as antioxidant-related metabolic markers in purple rice, while Zhu et al. [15] reported antioxidant metabolites in colored rice grains. Shawky et al. [16] identified 5H-5-methyl-6,7-dihydrocyclopenta[b]pyrazine, maltol, and epigallocatechin as metabolic markers associated with barley roasting. Wu et al. [17] employed LC-MS to characterize differential metabolites in Qingke-*Monascus* tea before and after in vitro digestion and reported higher antioxidant activity after digestion.

In summary, this study characterized 43 oat germplasm accessions and classified them into different activity groups according to the DPPH radical-scavenging activity and α-glucosidase inhibitory activity of their in vitro digestates. The differential metabolite composition between the high- and low-activity groups was subsequently analyzed using UPLC-MS/MS. A random forest model was employed to identify signature metabolites exhibiting significant variation in the targeted bioactivities. By integrating metabolomics with machine learning, this study provides a new strategy for the targeted development and efficient utilization of functional products.

## 2. Materials and Methods

### 2.1. Reagents and Instruments

Petroleum ether and anhydrous ethanol were obtained from Tianjin Tianli Chemical Reagent Co., Ltd. (Tianjin, China). Sodium dihydrogen phosphate, potassium sodium tartrate tetrahydrate, and glucose were obtained from Tianjin Comeo Chemical Reagent Co., Ltd. (Tianjin, China). Sodium chloride and 3,5-dinitrosalicylic acid were acquired from Sinopharm Chemical Reagent Co., Ltd. (Shanghai, China) and Tianjin Damao Chemical Reagent Factory (Tianjin, China), respectively. Phenol and methanol were procured from Jiangsu Pulisi Biotechnology Co., Ltd. (Nantong, China) and Tianjin Fuyu Fine Chemical Co., Ltd. (Tianjin, China), respectively. All chemicals were of analytical grade. α-Glucosidase, trypsin, and pepsin (biotechnology grade) were acquired from Shanghai Yuan ye Bio-Technology Co., Ltd. (Shanghai, China). The following instruments were employed in this work: a digital thermostatic stirring water bath (HH-6, Changzhou Hong ze Laboratory Technology Co., Ltd., Changzhou, China), a digital thermostatic shaking water bath (SHZ-82A, Changzhou Jin nan Instrument Manufacturing Co., Ltd., Changzhou, China), a benchtop high-speed centrifuge (HT185, Xiangyi Laboratory Instrument Development Co., Ltd., Changsha, China), and an ultrasonic cleaner (KQ3200DE, Kunshan Ultrasonic Instrument Equipment Co., Ltd., Kunshan, China). Additional instrumentation comprised a full-wavelength microplate reader (Multiskan GO, Thermo Fisher Scientific, Shanghai, China), a pH meter (pHS-3E, Shanghai Leici Instrument Co., Ltd., Shanghai, China), a heating magnetic stirrer (SN-DF-101T, Shang yi Scientific Instruments Co., Ltd., Shaoxing, China), and an electronic analytical balance (SQp, Sartorius Scientific Instruments Co., Ltd., Shanghai, China). Additional apparatus included a grinder (2500C, Dongguan Huatai Electric Appliance Co., Ltd., Dongguan, China), an SC-G automatic seed analyzer (Top Cloud-agri Technology Co., Ltd., Hangzhou, China), and an ultra-low temperature freezer (DW-CL, Hangzhou Cooling Equipment Co., Ltd., Hangzhou, China).

### 2.2. Plant Materials and Sample Preparation

A total of 43 oat accessions were sourced from seven distinct geographical regions, namely Hebei, Shanxi, Inner Mongolia, Gansu, Qinghai, and Jilin in China, as well as the United States. Following uniform processing, all samples were characterized for seed morphological traits. The geographical origins of the 43 oat accessions are detailed in Table 1. The collection effort was concentrated in northern China, consistent with the crop’s adaptation to cool climates. Hebei Province was the predominant source, contributing 22 accessions (51.2%), primarily from its major oat-producing region, Zhangjiakou. Eight accessions originated from Shanxi Province, whereas Jilin, Gansu, Qinghai, Inner Mongolia, and the United States each provided fewer than four. The germplasm collection consisted of 79% hull-less and 21% hulled oat accessions, as detailed in Figure S1. For each accession, one hundred fully developed seeds were randomly selected for morphological trait analysis using an SC-G automatic seed analyzer. The thousand-seed weight (TSW) was measured in triplicate using an electronic analytical balance. Prior to processing, hulled oat samples were mechanically debusked. Subsequently, following the removal of impurities, all samples were ground into a fine powder, sieved through a 40-mesh sieve, and stored at –20 °C for subsequent biochemical analysis.

### 2.3. In Vitro Simulated Gastrointestinal Digestion

The in vitro starch digestibility was evaluated using a modified method based on the protocols of Englyst et al. [18]. Briefly, a starch-equivalent sample of approximately 0.33 g was weighed into each of three 50 mL centrifuge tubes. Each centrifuge tube received 5 mL of deionized water. The mixtures were then heated in a boiling water bath for 20 min with intermittent vortexing at 5 min intervals, subsequently cooled to room temperature, and finally equilibrated at 37 °C for 10 min in a shaking water bath. For the 0 min control, 5 mL of sodium phosphate buffer (0.1 mol/L, pH 6.9) was added to a separate reaction tube. The remaining two tubes each received 5 mL of simulated gastric fluid. This fluid was prepared by dissolving 0.05 g of pepsin in 10 mL of a solution containing 2.4 mL of NaCl solution (0.4 g/L) and 7.6 mL of sodium phosphate buffer (0.1 mol/L, pH = 6.9), with the final pH adjusted to 2.0 using 2 mol/L HCl. Following a brief vortex, all tubes were incubated at 37 °C with continuous shaking at 180 rpm for 30 min. Subsequently, the pH in all tubes was adjusted to 6.9 with 4 mol/L NaOH. The control tube then received 5 mL of sodium phosphate buffer (0.1 mol/L, pH = 6.9), whereas the remaining tubes were supplemented with 5 mL of simulated intestinal fluid. The simulated intestinal fluid was prepared by dissolving 4 g of pancreatin (amylase activity: 7000 U/g) in 80 mL of distilled water. The resulting mixture was centrifuged at 1500 rpm for 10 min at room temperature. Subsequently, 54 mL of the pancreatin supernatant was mixed with 6 mL of glucoamylase solution (280 U/mL) and 4 mL of distilled water, giving a final volume of 64 mL. The final activities of glucoamylase and pancreatic amylase in the simulated intestinal fluid were 26.25 U/mL and 295.31 U/mL, respectively. All reaction mixtures were incubated in a shaking water bath at 37 °C and 180 rpm for 3 h. Following the digestion period, the samples were centrifuged at 8000× *g* for 20 min. The resulting supernatants were collected and stored at –80 °C pending subsequent analysis.

### 2.4. DPPH Radical-Scavenging Capacity

For each oat accession, three independent samples were prepared, and each sample underwent the digestion, extraction, and DPPH assay procedures separately. The digested supernatant was subjected to freeze-drying. Subsequently, 0.5 g of the resulting lyophilized powder was accurately weighed and extracted with 4 mL of 70% (*v*/*v*) aqueous methanol, corresponding to a solid-to-liquid ratio of 1:8 (*w*/*v*). The mixture was ultrasonicated at 50 °C for 15 min and then centrifuged at 10,000× *g* for 8 min. The resulting supernatant was collected, and the residual pellet was re-extracted following the same procedure. The two supernatants were combined and diluted with 70% (*v*/*v*) methanol to a final volume of 8 mL. The resulting extract corresponded to 62.5 mg of lyophilized digestate per mL, and the same extract concentration was used for all oat accessions. For the DPPH assay, 1 mL of the extract was mixed with 1 mL of DPPH solution, corresponding to 31.25 mg/mL of lyophilized digestate equivalent in the final reaction mixture. A 0.5 mmol/L DPPH solution was prepared by dissolving 0.01 g of DPPH in 50 mL of methanol. The mixture was incubated in darkness at room temperature for 20 min, and the absorbance was measured at 517 nm. DPPH radical-scavenging activity was evaluated using a fixed 20 min endpoint under identical reaction conditions for all samples. A sample blank containing the sample extract and methanol without DPPH was measured to correct for background absorbance. The DPPH radical-scavenging activity (%) was calculated from the absorbance values of the sample reaction, sample blank, and DPPH control according to Equation (1).

(1) $$\text{DPPH radical-scavenging activity (\%)}=(1-\frac{A_{s}-A_{s\beta }}{A_{c}})\times 100$$

where *A*_s_ is the absorbance of the sample reaction, *A*_sβ_ is the absorbance of the sample blank and *A*_c_ is the absorbance of the DPPH control.

### 2.5. α-Glucosidase Inhibitory Capacity

The α-glucosidase inhibitory activity was determined using a modified pNPG method [19]. The sample solution was prepared from the lyophilized oat digestate at a concentration equivalent to 62.5 mg/mL. Four reaction groups were prepared: the sample group (A), sample blank (A_0_), control group (B), and control blank (B_0_). For the sample group, 60 μL of PBS (0.1 mol/L, pH 6.8), 30 μL of sample solution (62.5 mg/mL), and 10 μL of α-glucosidase solution (0.8 U/mL) were mixed and incubated at 37 °C for 15 min. Subsequently, 10 μL of p-nitrophenyl-α-D-glucopyranoside (pNPG, 1 mM) was added, and the mixture was incubated at 37 °C for another 15 min. The concentration of lyophilized digestate equivalent in the 110 μL enzymatic reaction mixture was 17.05 mg/mL. The reaction was terminated by adding 80 μL of Na_2_CO_3_ solution (0.2 mol/L), and the absorbance was measured at 405 nm.

For the sample blank (A_0_), the α-glucosidase solution was replaced with 10 μL of PBS. For the control group (B), the sample solution was replaced with 30 μL of PBS. For the control blank (B_0_), both the sample solution and α-glucosidase solution were replaced with the corresponding volumes of PBS. The α-glucosidase inhibition rate was calculated according to Equation (2).

(2) $$\text{Inhibition rate (\%)}=(1-\frac{A-A_{0}}{B-B_{0}})\times 100\%$$

where *A* denotes the absorbance of the sample group, *A*_0_ represents the absorbance of the sample blank, *B* signifies the absorbance of the control group, and *B*_0_ corresponds to the absorbance of the control blank.

### 2.6. LC-MS Conditions

The samples were freeze-dried using a Scientz-100F lyophilizer and ground into powder at 30 Hz for 1.5 min using an MM400 grinder (Retsch). Approximately 50 mg of sample powder was weighed and mixed with 1200 μL of precooled 70% methanolic aqueous internal-standard extract. For samples weighing less than 50 mg, the volume of extractant was adjusted proportionally to 1200 μL per 50 mg of sample. The samples were vortexed for 30 s every 30 min for a total of six times. After centrifugation at 12,000 rpm for 3 min, the supernatant was collected, filtered through a 0.22 μm membrane, and transferred to an injection vial for UPLC-MS/MS analysis.

The sample extracts were analyzed using an UPLC-ESI-MS/MS system (UPLC, ExionLC™ AD, sciex.com.cn). The analytical conditions were as follows: UPLC: column, Agilent SB-C18 (1.8 μm, 2.1 mm × 100 mm); The mobile phase consisted of solvent A, pure water with 0.1% formic acid, and solvent B, acetonitrile with 0.1% formic acid. Sample measurements were performed with a gradient program that employed the starting conditions of 95% A, 5% B. Within 9 min, a linear gradient to 5% A, 95% B was programmed, and a composition of 5% A, 95% B was kept for 1 min. Subsequently, a composition of 95% A, 5.0% B was adjusted within 1.1 min and kept for 2.9 min. The flow velocity was set as 0.35 mL per minute; The column oven was set to 40 °C; The injection volume was 2 μL. The effluent was alternatively connected to an ESI-triple quadrupole-linear ion trap (QTRAP)-MS.

The ESI source operation parameters were as follows: source temperature 500 °C; ion spray voltage (IS) 5500 V (positive ion mode)/−4500 V (negative ion mode); ion source gas I (GSI), gas II (GSII), and curtain gas (CUR) were set at 50, 60, and 25 psi, respectively; collision-activated dissociation (CAD) was high. QQQ scans were acquired as MRM experiments with collision gas (nitrogen) set to medium. DP (declustering potential) and CE (collision energy) for individual MRM transitions were determined with further DP and CE optimization. A specific set of MRM transitions was monitored for each period according to the metabolites eluted within this period [20].

### 2.7. Statistical Analysis

Data are presented as mean ± standard deviation (SD), and the SD values were calculated from three independent replicates (*n* = 3). For activity-based grouping, the 43 oat accessions were ranked separately according to their DPPH radical-scavenging activity and α-glucosidase inhibitory activity. For each activity, the ten accessions with the highest values were assigned to Group I (high-activity group), the ten accessions with the lowest values were assigned to Group III (low-activity group), and the remaining 23 accessions were assigned to Group II (intermediate-activity group). The grouping was based on the ranking of the measured activity values rather than on an independent clustering algorithm. Principal component analysis (PCA), orthogonal partial least squares-discriminant analysis (OPLS-DA), and variable importance in projection (VIP) analyses were conducted using SIMCA software (version 14.1; Umetrics, Umea, Sweden). Random forest modeling was performed with R software (version 4.3.0; The R Foundation for Statistical Computing, Vienna, Austria). Heatmap and volcano plot analyses were generated using the online platform Bioinformatic (www.bioinformatics.com.cn). Data visualization was conducted using Origin software (version 2023; OriginLab Corporation, Northampton, MA, USA). Statistical differences were determined by Duncan’s test at a significance level of *p* < 0.05 using SPSS 26.0 (SPSS Inc., Chicago, IL, USA).

## 3. Results and Discussion

### 3.1. Geographic Distribution and Agronomic Traits of Oat Resources

The evaluation of oat accessions revealed significant variations in key agronomic traits and associated physiological activities. This study employed 43 oat accessions sourced from major oat-producing regions in China, such as the North, Northwest, and Northeast, as well as from international locations (Table 1). Prolonged natural selection and targeted breeding have produced distinct oat accessions with specific local adaptations across different geographical regions. For instance, Hebei Province has cultivated several characteristic cultivar series. These include the ‘Hua zao’ and ‘Hua wan’ series, distinguished by their short and long growth cycles, respectively; high-yielding naked oat series, such as ‘Bayou’ and ‘Zhang you’; and hulled oat series, including ‘Zhang yan’ and ‘Ji bai yan’. Similarly, Shanxi Province has developed its own characteristic series, such as ‘Jin yan’ and ‘Wei du You’. Notably, the accession ‘Jinyan 9’ (coded Y06 in this study) is classified as a hulled oat variety. Gansu province is primarily represented by its characteristic ‘Ning You’ and ‘Ding You’ series, alongside the hulled oat ‘Ding Yan’ series. Inner Mongolia is characterized by the cultivation of its regional series, including ‘Nei Yan’ and ‘Yan Ke’. Jilin Province’s oat production is centered on the ‘Bai Yan’ series. The oat accessions from Qinghai are predominantly hulled types, exemplified by the ‘Qing Yan’ series and sweet oat cultivars.

Representative grain samples from each geographical region were photographed under standardized lighting conditions to provide a comprehensive visual record (Figure S1). Visual inspection revealed discernible variations in kernel size and color among the different oat accessions. The analysis classified nine accessions as hulled oats, specifically Y01, Y06, Y12, Y17, Y24, Y28, Y29, Y30, and Y34. The remaining thirty-four accessions, constituting 79% of the total, were identified as naked oats. The predominance of naked oat accessions in this collection reflects the contemporary agricultural landscape in China, where they represent the primary cultivated type. Key morphological seed traits, including area, perimeter, roundness, length, width, and the length-to-width ratio, were quantified for each accession (Table 2). The thousand-kernel weight (TKW) of the oat accessions exhibited a range from 13.03 g to 38.49 g. This observed range falls within, but is situated at the lower end of, the spectrum of 16.17 to 52.75 g reported by Singh et al. [21]. Among the quantified morphological traits, thousand-kernel weight (TKW) demonstrated the highest coefficient of variation, which suggests substantial genetic diversity among the oat accessions. The accessions with the highest TKW values were Y34 (38.49 g) from Qinghai, Y12 (37.30 g) from Hebei, and Y28 (36.00 g) from Qinghai. Notably, all three accessions with high thousand-kernel weight are hulled oats. This observation aligns with the findings of Sykut-Domanska et al. [22], who attributed the characteristically greater kernel weight in hulled types to the additional mass of the hull itself. Owing to their elevated thousand-kernel weight, accessions Y34, Y28, and Y12 show strong potential for utilization as parental lines in breeding programs focused on yield enhancement.

Seed area and perimeter serve as key morphological traits for the identification of oat cultivars [23]. Among the 43 oat accessions examined, the seed length ranged from 6.76 to 13.22 mm, the width from 2.05 to 3.29 mm, the aspect ratio from 2.40 to 4.26, and the perimeter from 16.84 to 31.79 mm. Notably, three hulled oat accessions from the northwestern region, specifically Y17, Y28, and Y34, consistently exhibited the largest values for perimeter (27.53, 30.58, and 31.79 mm), length (11.67, 12.97, and 13.22 mm), and aspect ratio (4.23, 4.24, and 4.26). In contrast, the greatest kernel width was observed in the naked oat Y13 (3.29 mm) from Hebei, followed by the hulled accessions Y34 (3.19 mm) and Y12 (3.17 mm). The cultivated accessions in this study exhibited a broader range for both seed area, from 12.49 to 30.02 mm^2^, and roundness, from 0.24 to 0.42, compared to the wild population, which had an area range of 14.85 to 18.49 mm^2^ and a roundness range of 0.23 to 0.24. This observed morphological divergence is likely a consequence of local adaptation and selective breeding in the cultivated oat accessions. The hulled oat accession Y34 from Qinghai exhibited the largest seed area (30.02 mm^2^), followed by Y28 (28.96 mm^2^; Qinghai) and the naked oat Y29 (25.35 mm^2^; Hebei). These distinct morphological characteristics render these accessions highly distinguishable. Conversely, accessions Y11, Y31, Y13, and Y20, which exhibited the highest roundness values (ranging from 0.40 to 0.42), were representative of the typical geometric regularity of oat kernels.

**Figure 1:**
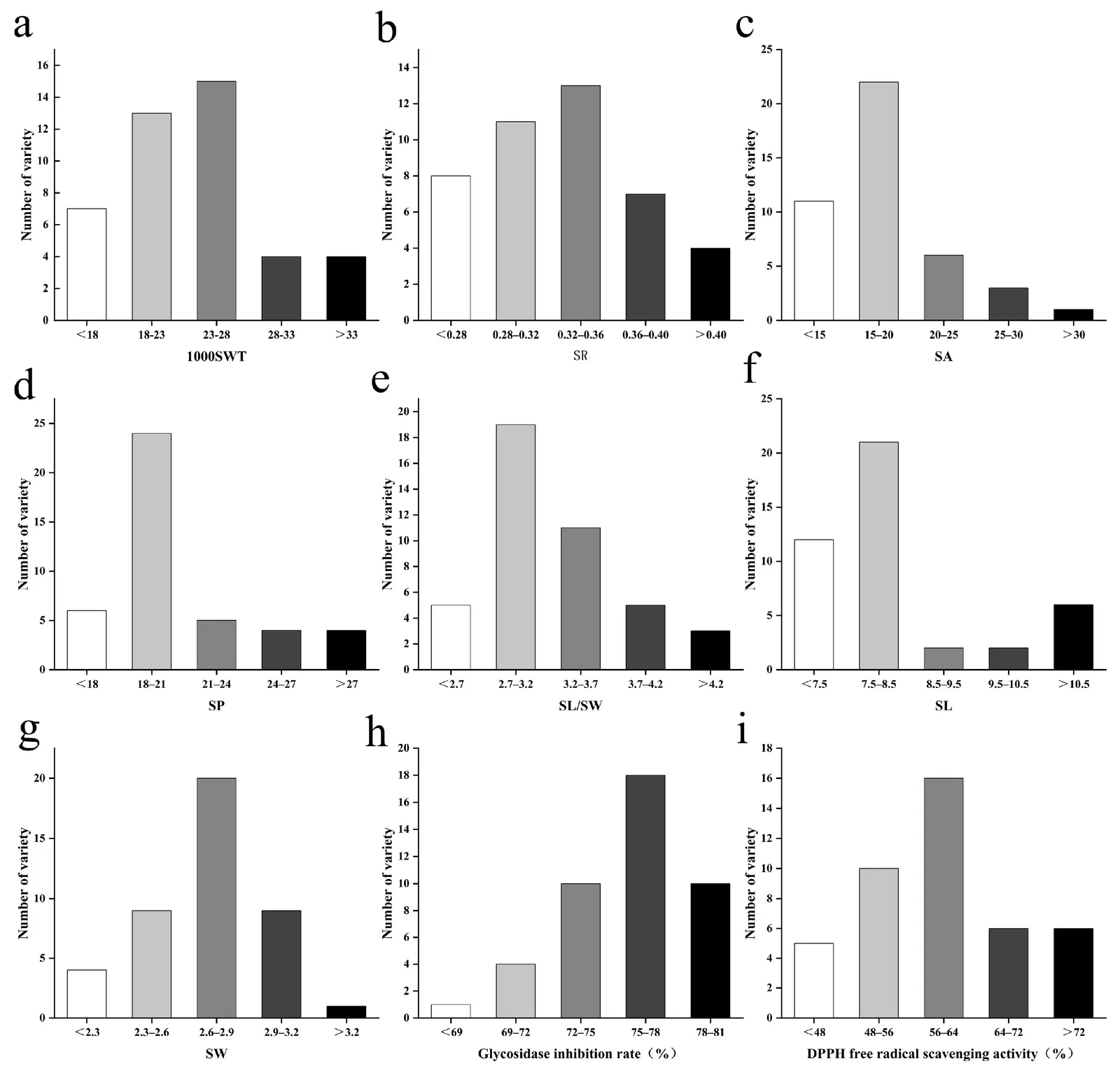
Frequency distributions of seed phenotypic traits and bioactivities among the 43 oat accessions. (**a**) Thousand-seed weight (1000 SWT); (**b**) seed roundness (SR); (**c**) seed area (SA); (**d**) seed perimeter (SP); (**e**) seed length-to-width ratio (SL/SW); (**f**) seed length (SL); (**g**) seed width (SW); (**h**) α-glucosidase inhibition rate; and (**i**) DPPH radical-scavenging activity. The *y*-axis represents the number of oat accessions within each predefined interval.

Frequency distribution plots were generated to visualize the variation in key seed morphological traits among the 43 oat accessions (Figure 1). All measured seed traits exhibited an approximately normal distribution, which supports the statistical reliability of the data. The unimodal distributions, characterized by a central clustering of accessions with fewer than 28% at the extremes, indicate substantial phenotypic diversity within the oat panel. This structured variation, particularly the identification of accessions at the distributional extremes, established a robust foundation for subsequent physiological and functional analyses.

### 3.2. Physiological Activity and Activity-Based Grouping of Oat Digestates

Significant variation was observed in the physiological activity of the digested oat samples among the different accessions. The physiological activity of the digested samples was quantified for all 43 oat accessions (Table 2). In the present study, the DPPH radical-scavenging activity of the digested oat samples ranged from 40.00% to 78.72%. In comparison, Saad et al. [24] reported DPPH radical-scavenging values ranging from 17.00% to 73.30% for other cereal by-products. Among the oat accessions evaluated in this study, Y15 showed the highest DPPH radical-scavenging activity (78.72%), whereas Y35 showed the lowest activity (40.00%). Y19 and Y20 also showed relatively high DPPH radical-scavenging activities. These results indicate considerable variation in DPPH radical-scavenging activity among the digested oat samples. Similar differences in antioxidant-related activities among oat accessions have also been reported by Ma et al. [8]. The potential for blood glucose regulation was evaluated by measuring the α-glucosidase inhibitory activity of the digested oat samples. The α-glucosidase inhibitory activity across the 43 oat accessions ranged from 60.30% to 80.17%, a range consistent with that reported for other cereals by Wu et al. [17]. Furthermore, the significant differences in α-glucosidase inhibitory activity observed among the oat accessions in this study aligned with the reported variability in the hypoglycemic capacity of endogenous compounds in naked oats, as documented by Wei et al. [25]. This investigation also identified notable disparities in α-glucosidase inhibitory activity. Specifically, accessions Y04, Y12, and Y26 demonstrated the most potent activity, which was approximately 75% higher than that of the lowest-performing accession, Y03. This finding confirms the substantial variation in the blood glucose-regulating potential among oat accessions and thereby corroborates previous reports. The distribution of these physiological activities is shown in the frequency distribution charts in Figure 1h,i. The data for both traits approximated a normal distribution, as evidenced by the majority of accessions clustering around intermediate values and fewer than 23% positioned at the distributional extremes. These extreme-value accessions are particularly valuable for future research aimed at elucidating the determinants of high antioxidant and hypoglycemic activity.

**Figure 2:**
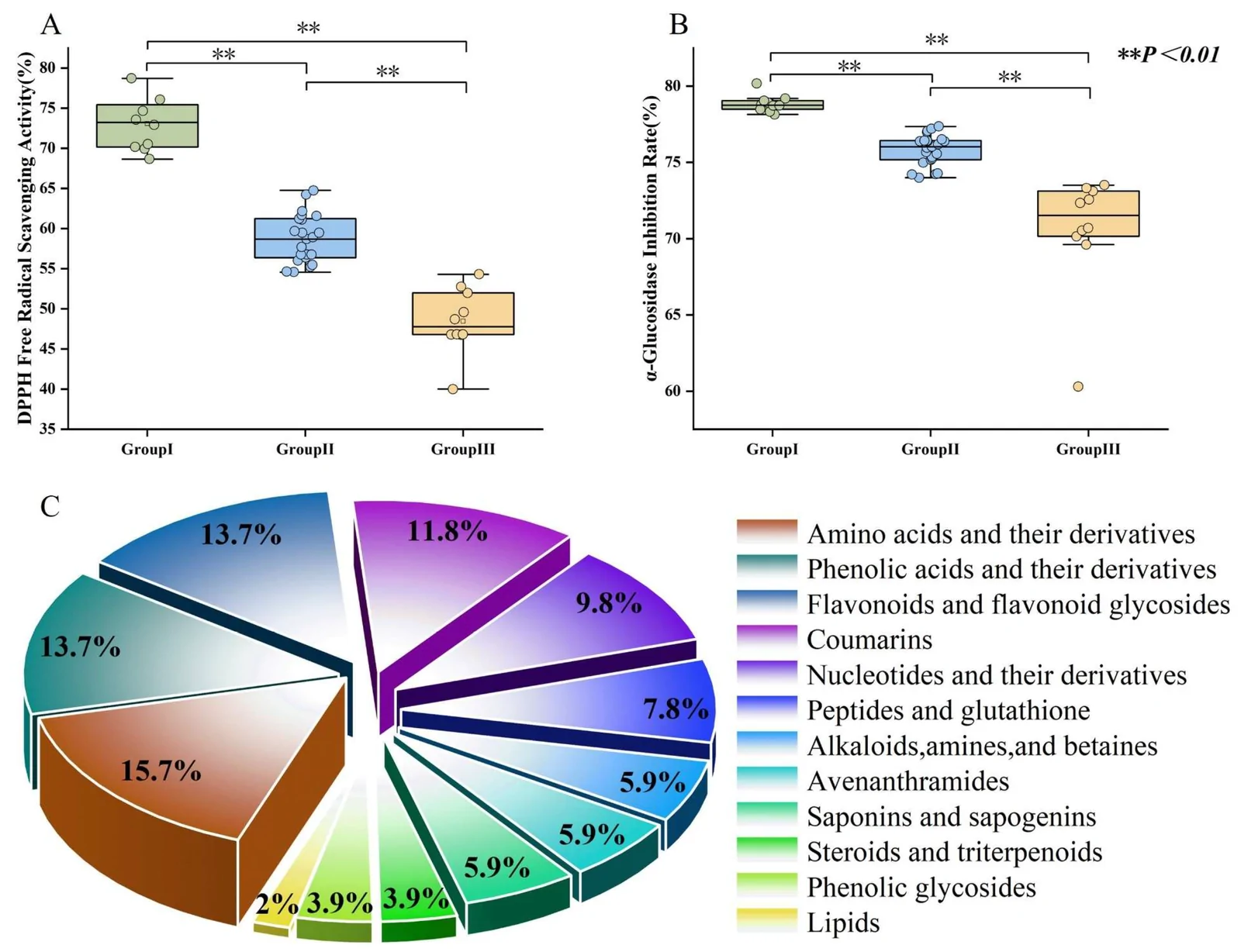
Bioactivity-based classification of oat accessions and chemical classification of metabolites. (**A**) DPPH radical-scavenging activity of oat accessions classified into high-, intermediate-, and low-activity groups according to their ranked activity values. (**B**) α-Glucosidase inhibitory activity of oat accessions classified using the same ranking-based procedure. The boxes indicate the interquartile range, and the horizontal line within each box represents the median. Statistical differences among groups were determined by one-way analysis of variance followed by Duncan’s multiple range test. Brackets indicate pairwise comparisons, and ** denotes *p* < 0.01. (**C**) Distribution of 72 annotated metabolites among 12 chemical classes.

Antioxidant activity and blood glucose regulation are important functional properties of oats [26,27]. Based on the measured DPPH radical-scavenging activity and α-glucosidase inhibitory activity, the 43 oat accessions were ranked separately and divided into three activity groups (Figure 2A,B). For DPPH radical-scavenging activity (Figure 2A), the ten accessions with the highest DPPH radical-scavenging capacity, namely Y15, Y19, Y20, Y36, Y39, Y16, Y40, Y18, Y30, and Y43, were assigned to Group I, representing the high-activity group. Because of their high DPPH radical-scavenging activity, these accessions may be useful for developing functional oat foods associated with the prevention of cardiovascular and cerebrovascular diseases and the delay of aging [28,29]. They may also be used as parental materials in breeding programs aimed at improving antioxidant activity. The ten accessions with the lowest DPPH radical-scavenging activity, including Y21, Y22, Y33, Y34, Y35, Y17, Y28, Y27, Y13, and Y03, were assigned to Group III, representing the low-activity group, whereas the remaining accessions were placed in the intermediate Group II. For α-glucosidase inhibitory activity (Figure 2B), Y12, Y19, Y04, Y26, Y41, Y36, Y31, Y23, Y14, and Y42 showed the highest inhibitory activity and were assigned to Group I. These accessions may be useful for developing oat products intended for the prevention and treatment of diabetes and may be regarded as potential natural antidiabetic materials [30]. They may also be used as parental materials in breeding programs aimed at improving α-glucosidase inhibitory activity. The ten accessions with the lowest α-glucosidase inhibitory activity, including Y01, Y02, Y03, Y22, Y24, Y08, Y38, Y43, Y05, and Y17, were assigned to Group III, whereas the remaining accessions formed the intermediate Group II. The box plots showed highly significant differences in antioxidant activity and α-glucosidase inhibitory activity among the three groups, with the clearest differences observed between Groups I and III. These results clearly reflected the separation among the three activity groups.

### 3.3. Identification and Analysis of Metabolites in Digestive Products from Different Oat Accessions

To further investigate the chemical basis of the observed differences in bioactivity, untargeted metabolomic profiling was conducted in both positive- and negative-ion modes using ultra-performance liquid chromatography–tandem mass spectrometry (UPLC–MS/MS). Metabolites detected in each ionization mode were annotated against the in-house Metware Database (MWDB), and the data from the two modes were merged. A total of 72 metabolites were identified, fewer than the 107 metabolites reported by Aly Castillo et al. [31] in oat -based beverages. This discrepancy may mainly reflect differences in sample source and processing procedures. The identities of the 72 metabolites were assigned through comparison of their mass spectral data with authentic standards and published reference data; detailed identification information is provided in Supplementary Table S1. Following the classification method of Wu et al. [17], the identified metabolites were grouped into 12 classes. Figure 2C shows their distribution: amino acids and derivatives (15.7%), phenolic acids and derivatives (13.7%), flavonoids and flavonoid glycosides (13.7%), coumarins (11.8%), nucleotides and derivatives (9.8%), small peptides and glutathione (7.8%), alkaloids and betaines (5.9%), avenanthramides (5.9%), saponins and sapogenins (5.9%), sterols and triterpenoids (3.9%), phenolic glycosides (3.9%), and lipids (2.0%).

**Figure 3:**
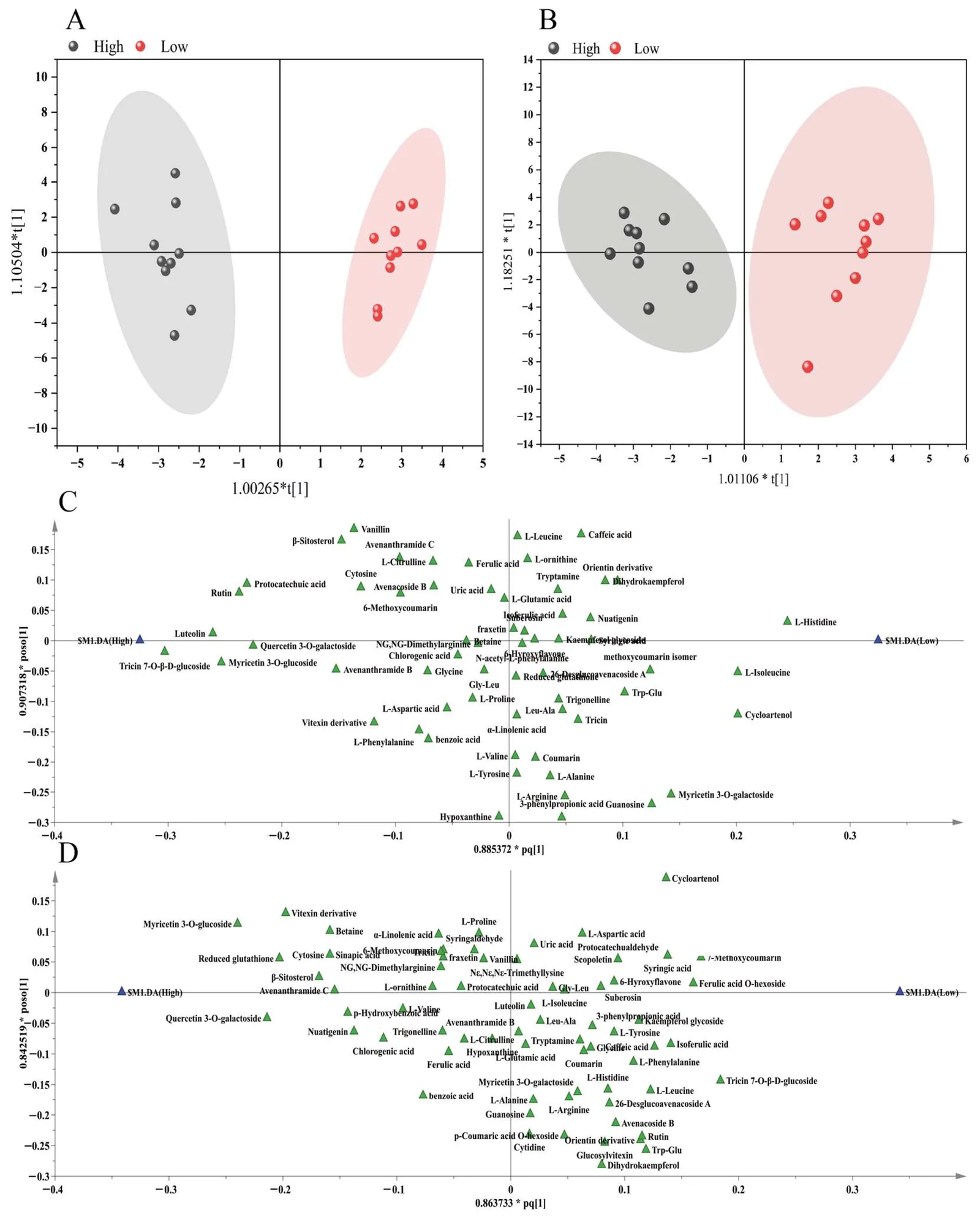
OPLS-DA score and loading plots of metabolite profiles in the in vitro digestion supernatants of oat accessions with contrasting bioactivities. (**A**) OPLS-DA score plot comparing oat samples with high and low DPPH radical-scavenging activity. (**B**) OPLS-DA score plot comparing oat samples with high and low α-glucosidase inhibitory activity. (**C**) OPLS-DA loading plot corresponding to the DPPH radical-scavenging activity model shown in panel A. (**D**) OPLS-DA loading plot corresponding to the α-glucosidase inhibitory activity model shown in panel B. Each point in panels (**A**,**B**) represents one biological sample, and the shaded ellipses indicate the 95% confidence regions. The labeled triangles in panels (**C**,**D**) represent annotated metabolites. High and Low denote the high- and low-activity groups, respectively.

Orthogonal partial least-squares discriminant analysis (OPLS-DA) is a supervised multivariate method that provides greater discriminatory power than principal component analysis (PCA) and is suitable for detecting differences in metabolite profiles among varieties [32]. In this study, OPLS-DA was used to further examine differences in metabolite profiles between the high- and low-activity groups for antioxidant activity and α-glucosidase inhibitory activity in 20 oat accessions. The OPLS-DA score plot for DPPH radical-scavenging activity shows clear separation between the 10 high-activity accessions and the 10 low-activity accessions (Figure 3A). The corresponding loading plot shows that individual metabolites differ in their contributions to the separation of the two groups (Figure 3C). Tricin 7*-O*-β-D-glucoside, protocatechuic acid, rutin, luteolin, myricetin 3*-O*-glucoside, and quercetin 3*-O*-galactoside are mainly associated with the high-DPPH group, whereas L-histidine and cycloartenol are located primarily on the positive side of the loading plot and are associated with the low-DPPH group. The OPLS-DA score plot for α-glucosidase inhibitory activity also shows clear separation between the 10 high-activity accessions and the 10 low-activity accessions. The corresponding loading plot indicates that individual metabolites differ in their contributions to the separation of the groups with high and low α-glucosidase inhibitory activity (Figure 3D). Myricetin 3*-O*-glucoside, vitexin derivative, quercetin 3*-O*-galactoside, reduced glutathione, and β-sitosterol are mainly associated with the high-inhibition group, whereas ferulic acid O-hexoside, tricin 7*-O*-β-D-glucoside, and 7-methoxycoumarin are mainly associated with the low-inhibition group. These results indicate marked differences in metabolite profiles among the oat accessions.

### 3.4. Analysis of Differential Metabolites in Digestive Products from Different Oat Accessions

**Figure 4:**
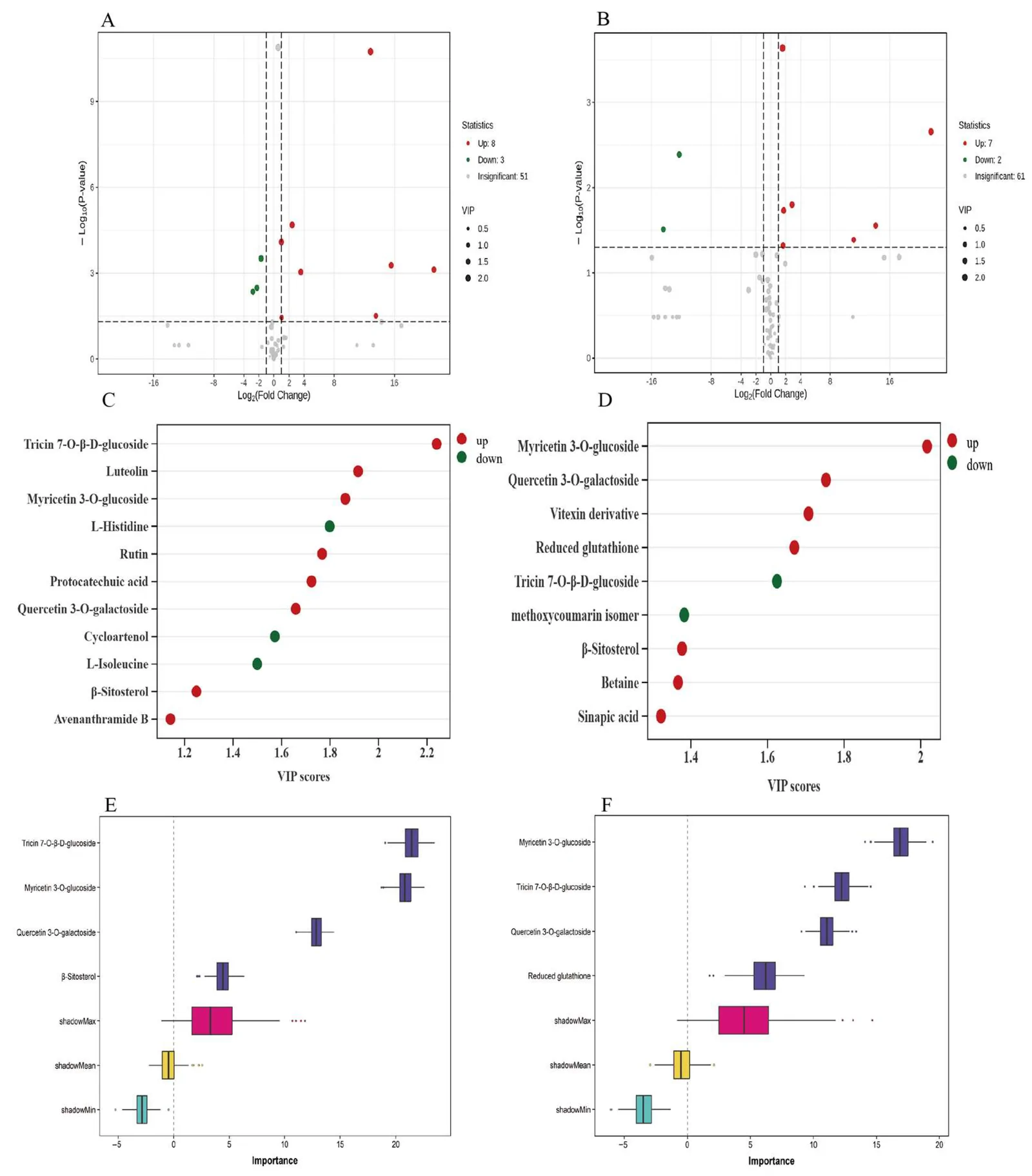
Differential metabolite screening and machine-learning-based selection of candidate metabolite markers associated with DPPH radical-scavenging and α-glucosidase inhibitory activities in oat accessions. (**A**,**B**) Volcano plots showing differences in metabolite abundance between the high- and low-activity groups for DPPH radical-scavenging activity (**A**) and α-glucosidase inhibitory activity (**B**). Red, green, and gray points indicate metabolites with higher abundance, lower abundance, and no significant difference, respectively, in the high-activity group relative to the low-activity group. Dashed lines indicate the fold-change and *p*-value thresholds used for differential metabolite screening, and point size represents the variable importance in projection (VIP) score. (**C**,**D**) VIP score plots of differential metabolites associated with DPPH radical-scavenging activity (**C**) and α-glucosidase inhibitory activity (**D**). Red and green points indicate metabolites with higher and lower abundance, respectively, in the high-activity group relative to the low-activity group. (**E**,**F**) Boruta feature-importance plots generated from random forest models for DPPH radical-scavenging activity (**E**) and α-glucosidase inhibitory activity (**F**). Metabolite importance distributions are shown relative to the shadow variables, including shadow Max, shadow Mean, and shadow Min. Points beyond the whiskers indicate outliers.

The main objective of this study was to investigate the metabolic basis of differences in physiological activity. The high- and low-activity groups were compared using volcano plot analysis, which enables the rapid screening of differential metabolites with potential biological relevance [33]. Figure 4A,C shows the metabolites that differed significantly between the groups in relation to antioxidant activity. Eleven differential metabolites were identified. Tricin 7*-O*-β-D-glucoside, luteolin, myricetin 3*-O*-glucoside, rutin, protocatechuic acid, quercetin 3*-O*-galactoside, β-sitosterol, and avenanthramide B were significantly upregulated, whereas L-histidine, cycloartenol, and L-isoleucine were significantly downregulated. Classification of these metabolites showed that the compounds enriched in the high-DPPH group I were mainly flavonoids and flavonoid glycosides, phenolic acids, and avenanthramides. This distribution suggested that these phenolic metabolites may contribute to differences in DPPH radical-scavenging activity among oat accessions.

Similar changes were reported in previous oat studies. Huang et al. [34] found that avenanthramides and flavonoid glycosides gradually accumulated during oat germination, accompanied by increases in DPPH and ABTS radical-scavenging activities. Pichler et al. [35] also reported increases in avenanthramide and phenolic acid contents, together with increased antioxidant activity after germination. Menga et al. [36] observed significant positive correlations of DPPH and ABTS antioxidant activities with avenanthramides and phenolic acids, including caffeic acid, p-coumaric acid, and ferulic acid, among different oat accessions. Kumar et al. [37] reported that phenolic acids possessed strong antioxidant properties. These observations are consistent with the significantly higher relative levels of flavonoid glycosides, phenolic acids, and avenanthramide B in the high-DPPH Group in the present study. Flavonoids and their glycosides, phenolic acids, and avenanthramides may represent major metabolic components associated with differences in DPPH radical-scavenging activity among oat accessions. Compared with low-activity Group III, high-DPPH Group I had significantly lower relative levels of L-histidine, L-isoleucine, and cycloartenol. Previous oat studies have mainly classified L-histidine and L-isoleucine as metabolites involved in protein and amino acid metabolism [38], whereas cycloartenol has been described as a precursor of phytosterol biosynthesis [39]. Direct evidence for a negative association between these metabolites and antioxidant activity in oats remains limited. The present results indicate only that these three metabolites were negatively associated with high antioxidant activity and may be regarded as candidate metabolites requiring further validation.

Volcano plot analysis was used to identify differential metabolites between the groups with high and low α-glucosidase inhibitory activity (Figure 4B,D). Seven differential metabolites were significantly upregulated in high-activity Group I relative to low-activity Group III. These included three compounds classified as flavonoids or flavonoid glycosides—myricetin 3*-O*-glucoside, quercetin 3*-O*-galactoside, and a vitexin derivative—as well as reduced glutathione, the phytosterol β-sitosterol, the alkaloid betaine, and the phenolic acid sinapic acid. Previous studies reported that both free and bound bioactive fractions of naked oats exhibited α-glucosidase inhibitory activity. Oat phenolic fractions rich in flavonoids, flavonoid glycosides, and phenolic acids were reported to inhibit α-glucosidase through competitive or mixed mechanisms [26]. Bioactive peptides generated by oat protein hydrolysis were also reported to inhibit α-glucosidase [31]. The enrichment of myricetin 3*-O*-glucoside, quercetin 3*-O*-galactoside, the vitexin derivative, and sinapic acid in Group I may be associated with its higher α-glucosidase inhibitory activity. Direct evidence for α-glucosidase inhibition by reduced glutathione, β-sitosterol, and betaine in oat-derived systems remains limited. At present, these compounds are best regarded as candidate metabolites associated with high inhibitory activity. Tricin 7*-O*-β-D-glucoside and 7-methoxycoumarin were significantly decreased in Group I, but direct evidence linking either compound to α-glucosidase inhibitory activity remains lacking. These two compounds are more appropriately considered characteristic metabolites of the low-activity group.

### 3.5. Screening Key Differential Metabolites Using a Random Forest Model

The random forest algorithm is widely used in data analysis and machine learning because of its high predictive accuracy, robustness, interpretability, and ability to handle high-dimensional data [40,41,42]. In this study, separate random forest models were constructed for DPPH radical-scavenging capacity and α-glucosidase inhibitory capacity using the corresponding high- and low-activity groups (Figure 4E,F). Metabolites with relatively high predictive value were selected according to their variable importance. Previous studies have shown that metabolomics combined with random forest analysis can be used to classify nutritional and functional traits in cereal crops and to screen candidate markers. Tiozon et al. [43] used random forest models to classify the nutritional and antioxidant properties of pigmented rice, and the model achieved an accuracy of 100% for the high-antioxidant-activity group. Shinn et al. [44] constructed a prediction model for whole-grain oat intake based on metabolite features and achieved a maximum accuracy of 89%, indicating that combinations of metabolites can be used to identify oat-related characteristics.

In the DPPH radical-scavenging capacity model, tricin 7*-O*-β-D-glucoside, myricetin 3*-O*-glucoside, quercetin 3*-O*-galactoside, and β-sitosterol showed high variable importance and were selected as candidate predictors and indicators of the DPPH radical-scavenging capacity of oat digestates. The model achieved a mean accuracy of 100%. The candidate metabolites selected by the random forest model were further examined in relation to previous studies. Tricin 7-*O*-β-D-glucoside isolated from Setaria viridis showed strong DPPH radical-scavenging activity and significantly inhibited lipid peroxidation in rat liver microsomes [45]. Myricetin 3-*O*-glucoside was also identified among phenolic compounds with radical-scavenging properties using an HPLC-DPPH post-column method [46]. Quercetin 3-*O*-galactoside, also known as hyperoside, was isolated and evaluated using DPPH, β-carotene bleaching, and reducing power assays and showed antioxidant activity in vitro [47]. These reports are consistent with the higher abundance of these flavonoid glycosides observed in the high-DPPH group in the present study. In contrast, β-sitosterol should not be interpreted as a direct DPPH-scavenging antioxidant. Previous studies have shown that β-sitosterol can modulate endogenous antioxidant defenses in macrophages by affecting the GSH/GSSG balance and the activities of manganese superoxide dismutase (Mn-SOD) and glutathione peroxidase (GPx) [48]. In addition, phytosterols are susceptible to oxidation and can form phytosterol oxidation products during storage or processing [49]. Therefore, the selection of β-sitosterol by the random forest model should be interpreted as evidence of its association with the high-DPPH phenotype rather than as evidence that β-sitosterol directly scavenges DPPH radicals.

In the α-glucosidase inhibitory capacity model, myricetin 3*-O*-glucoside, quercetin 3*-O*-galactoside, and reduced glutathione also showed high variable importance and were identified as candidate predictors and indicators of the α-glucosidase inhibitory capacity of oat digestates. The model achieved an accuracy of 90%. Myricetin 3-*O*-glucoside and quercetin 3-*O*-galactoside were included in both models, suggesting that these two flavonoid glycosides may serve as shared metabolic markers of variation in antioxidant activity and α-glucosidase inhibitory capacity among oat digestates. Previous studies also provide some support for the candidate markers identified for α-glucosidase inhibitory activity. Wasai et al. reported that myricetin 3-*O*-glucoside inhibited α-glucosidase in a cell-free assay and was present at relatively high levels in the green tea cultivar Sunrouge, which showed stronger glucose-lowering potential [50]. Quercetin 3-*O*-galactoside has also been evaluated directly for α-glucosidase inhibition. Yan et al. purified this compound from Chinese bayberry and reported an IC_50_ value of 556.4 μg/mL against α-glucosidase [51]. These findings are consistent with the selection of these two flavonoid glycosides as candidate markers in the present model. Reduced glutathione was also selected by the random forest model. It is a major non-enzymatic antioxidant involved in cellular redox homeostasis and functions both as a direct antioxidant and as a cofactor for glutathione-dependent enzymes such as glutathione peroxidase [52]. However, direct evidence showing that reduced glutathione inhibits α-glucosidase is still limited. Therefore, in the present study, reduced glutathione is more reasonably regarded as a metabolite associated with the high-inhibition phenotype rather than as a compound directly responsible for α-glucosidase inhibition.

This study still has some limitations. The in vitro digestion method provided the same experimental conditions for all oat accessions, but this static system cannot fully reproduce the dynamic process of human gastrointestinal digestion [53]. In addition, the intestinal permeability, absorption, and bioavailability of the identified metabolites were not further evaluated using intestinal cell models or in vivo experiments. Therefore, the metabolites detected after digestion only reflect their release under the present experimental conditions and do not necessarily indicate that they can be efficiently absorbed in humans [54]. The effect of the oat food matrix was also not evaluated separately. Proteins, lipids, starch, and dietary fiber may influence the release, stability, and bioaccessibility of bioactive compounds during digestion [55]. The DPPH assay used in this study was based on a fixed 20 min endpoint. Because the reaction was not monitored over time, the present data do not provide information on the rate of radical scavenging or confirm that all antioxidant constituents reached a steady state within 20 min. Therefore, the DPPH results should be interpreted as comparative radical-scavenging responses measured under the same endpoint conditions rather than as a kinetic evaluation of antioxidant effectiveness. Future studies should include time-course measurements to distinguish rapid and slow radical-scavenging responses. Moreover, the candidate markers selected by the random forest models mainly indicate associations with DPPH radical-scavenging and α-glucosidase inhibitory activities rather than direct causal effects. Further studies using intestinal cell models and in vivo experiments are needed to confirm the biological effects and bioavailability of these candidate metabolites. Their activities should also be evaluated in oat-based food formulations to determine whether the effects observed under the present experimental conditions can be maintained in more realistic food systems.

## 4. Conclusions

This study systematically evaluated the agronomic traits of 43 oat accessions from different geographic origins, together with the DPPH radical-scavenging and α-glucosidase inhibitory activities of their in vitro digestates. Significant differences among accessions were observed in the measured agronomic traits and bioactivities. Metabolomic profiling using ultra-performance liquid chromatography–tandem mass spectrometry (UPLC–MS/MS) identified 72 metabolites in the digestates, mainly comprising amino acids and derivatives, phenolic acids and derivatives, and flavonoids and flavonoid glycosides. Orthogonal partial least squares discriminant analysis (OPLS-DA) clearly separated the high- and low-activity groups. Random forest analysis identified tricin 7-*O*-β-D-glucoside, myricetin 3-*O*-glucoside, quercetin 3-*O*-galactoside, and β-sitosterol as candidate markers associated with DPPH radical-scavenging activity, and myricetin 3-*O*-glucoside, quercetin 3-*O*-galactoside, and reduced glutathione as candidate markers associated with α-glucosidase inhibitory activity. β-Sitosterol was retained as a statistical marker of the high-DPPH phenotype rather than being interpreted as a direct radical-scavenging antioxidant. This study integrated metabolomics with machine learning for the first time to systematically characterize differences in the bioactive metabolite profiles of oat digestates. The findings provide a theoretical basis for the targeted development, screening, and value-added utilization of functional oat products.

## Figures and Tables

**Table 1:** Names, hull types, and geographic origins of the 43 oat accessions.

Accession No.	Accession Name	Hull Type	Geographic Origin	Accession No.	Accession Name	Hull Type	Geographic Origin
Y01	Menglong	H	United States	Y23	Zhangyou 4	N	Hebei
Y02	Neiyan 5	N	Inner Mongolia	Y24	Zhangyan 5	H	Hebei
Y03	Jinyan 14	N	Shanxi	Y25	Zhangyou 6	N	Hebei
Y04	Bayou 8	N	Hebei	Y26	Zhangyou 12	N	Hebei
Y05	Zhangyou 9	N	Hebei	Y27	Jinyan 17	N	Shanxi
Y06	Jinyan 9	H	Shanxi	Y28	Qingyin 1	H	Qinghai
Y07	Yanke 1	N	Inner Mongolia	Y29	Zhangyan 4	H	Hebei
Y08	201229-1-1	N	Hebei	Y30	Zhangyan 3	H	Hebei
Y09	Bayou 18	N	Hebei	Y31	Jinyan 13	N	Shanxi
Y10	Weiduyou 5	N	Shanxi	Y32	Baiyan 8	N	Jilin
Y11	Huazao 2	N	Hebei	Y33	Yanke 2	N	Inner Mongolia
Y12	Jibaiyan 3	H	Hebei	Y34	Sweet Oat	H	Qinghai
Y13	Huawan 6	N	Hebei	Y35	Zhangyou 2	N	Hebei
Y14	Zhangyou 3	N	Hebei	Y36	Bayou 14	N	Hebei
Y15	Ningyou 1	N	Gansu	Y37	Bayou 5	N	Hebei
Y16	Jinyan 1	N	Shanxi	Y38	Bayou 6	N	Hebei
Y17	Dingyan 2	H	Gansu	Y39	Baiyan 11	N	Jilin
Y18	Zhangyou 5	N	Hebei	Y40	Bayou 9	N	Hebei
Y19	Jinyan 8	N	Shanxi	Y41	Zhangyou 7	N	Hebei
Y20	Baiyan 18	N	Jilin	Y42	Dingyou 1	N	Gansu
Y21	Jinyan 18	N	Shanxi	Y43	Bayou 3	N	Hebei
Y22	Baiyan 15	N	Jilin				

Note: H, hulled oat; N, naked oat.

**Table 2:** Seed phenotypic traits and bioactivities of 43 oat accessions.

No.	1000 SWT (g)	SA (mm^2^)	SP (mm)	SL/SW	SL (mm)	SW (mm)	SR (mm)	DPPH (%)	α-Glucosidase (%)
Y01	27.43 ± 0.87	23.45 ± 1.24	24.95 ± 1.56	3.49 ± 0.15	10.35 ± 0.15	3.02 ± 0.25	0.29 ± 0.03	58.7 ± 5.2	70.15 ± 5.6
Y02	23.66 ± 1.23	15.91 ± 0.95	18.83 ± 1.89	2.96 ± 0.12	7.76 ± 0.12	2.65 ± 0.28	0.34 ± 0.04	62.18 ± 3.8	70.7 ± 3.8
Y03	25.52 ± 0.56	17.31 ± 1.68	21.57 ± 1.24	2.92 ± 0.18	8.23 ± 0.18	2.91 ± 0.22	0.36 ± 0.02	48.71 ± 6.7	60.3 ± 6.2
Y04	22.55 ± 1.09	15.96 ± 1.03	19.52 ± 2.15	3.29 ± 0.11	8.09 ± 0.11	2.49 ± 0.29	0.3 ± 0.05	64.76 ± 4.5	79.05 ± 4.3
Y05	23.51 ± 0.72	15.58 ± 1.82	19.52 ± 1.67	2.97 ± 0.16	7.74 ± 0.16	2.66 ± 0.23	0.35 ± 0.03	64.25 ± 7.3	72.56 ± 5.9
Y06	30.99 ± 1.38	22.36 ± 1.35	22.83 ± 1.38	3.06 ± 0.09	9.42 ± 0.09	3.06 ± 0.26	0.33 ± 0.04	61.12 ± 3.2	75.57 ± 3.2
Y07	17.71 ± 0.41	15.57 ± 0.89	19.69 ± 1.97	3.46 ± 0.19	8.25 ± 0.19	2.39 ± 0.21	0.29 ± 0.02	58.93 ± 5.9	75.25 ± 4.9
Y08	23.91 ± 0.95	17.72 ± 1.51	20.24 ± 1.45	3.17 ± 0.13	8.40 ± 0.13	2.67 ± 0.27	0.31 ± 0.05	61.57 ± 4.1	73.51 ± 6.7
Y09	14.51 ± 1.17	13.53 ± 1.17	19.34 ± 2.08	4.03 ± 0.17	8.22 ± 0.17	2.07 ± 0.24	0.24 ± 0.03	55.23 ± 6.3	76.99 ± 3.5
Y10	23.92 ± 0.63	16.21 ± 1.74	20.07 ± 1.73	3.47 ± 0.14	8.46 ± 0.14	2.45 ± 0.20	0.29 ± 0.04	56.38 ± 7.8	74.24 ± 5.1
Y11	24.77 ± 0.35	16.47 ± 0.98	19.49 ± 1.12	2.60 ± 0.10	7.49 ± 0.10	2.95 ± 0.28	0.40 ± 0.02	55.48 ± 3.5	76.38 ± 6.4
Y12	37.30 ± 1.40	24.86 ± 1.42	25.11 ± 2.22	3.29 ± 0.2	10.38 ± 0.2	3.17 ± 0.25	0.31 ± 0.05	57.71 ± 5.6	79.19 ± 4.6
Y13	31.12 ± 0.89	19.01 ± 1.21	21.54 ± 1.59	2.53 ± 0.15	7.92 ± 0.15	3.29 ± 0.29	0.41 ± 0.03	49.59 ± 4.9	77.07 ± 5.3
Y14	14.94 ± 1.02	12.75 ± 1.88	17.84 ± 1.81	3.12 ± 0.12	7.18 ± 0.12	2.33 ± 0.23	0.32 ± 0.04	54.65 ± 6.9	78.32 ± 3.9
Y15	30.12 ± 0.51	20.7 ± 1.09	22.83 ± 1.32	3.34 ± 0.16	9.47 ± 0.16	2.86 ± 0.26	0.30 ± 0.02	78.72 ± 7.1	74.28 ± 6.1
Y16	23.60 ± 1.28	16.48 ± 1.63	19.34 ± 2.01	3.07 ± 0.18	7.98 ± 0.18	2.63 ± 0.22	0.33 ± 0.05	72.93 ± 3.9	76.03 ± 4.2
Y17	28.69 ± 0.76	23.84 ± 0.85	27.53 ± 1.63	4.23 ± 0.13	11.64 ± 0.13	2.78 ± 0.27	0.24 ± 0.03	54.31 ± 5.4	72.34 ± 5.7
Y18	17.69 ± 0.99	14.01 ± 1.39	19.00 ± 1.49	3.24 ± 0.11	7.64 ± 0.11	2.42 ± 0.24	0.31 ± 0.04	70.18 ± 4.3	75.96 ± 3.1
Y19	20.94 ± 1.33	15.45 ± 1.57	19.35 ± 1.92	3.41 ± 0.19	8.09 ± 0.19	2.42 ± 0.21	0.29 ± 0.02	76.07 ± 6.5	78.91 ± 4.8
Y20	24.4 ± 0.47	14.99 ± 1.12	16.99 ± 2.18	2.4 ± 0.09	6.76 ± 0.09	2.80 ± 0.20	0.42 ± 0.05	75.44 ± 8.0	76.19 ± 6.9
Y21	13.03 ± 0.68	13.19 ± 1.79	19.21 ± 1.28	4.02 ± 0.14	8.16 ± 0.14	2.05 ± 0.28	0.24 ± 0.03	46.82 ± 3.1	74.00 ± 3.7
Y22	23.27 ± 1.12	15.35 ± 1.05	17.84 ± 1.77	2.64 ± 0.17	7.17 ± 0.17	2.75 ± 0.25	0.38 ± 0.04	46.78 ± 5.8	70.53 ± 5.4
Y23	13.37 ± 0.39	12.49 ± 1.48	17.53 ± 1.51	3.26 ± 0.10	7.17 ± 0.10	2.23 ± 0.29	0.31 ± 0.02	54.59 ± 4.7	78.48 ± 6.5
Y24	33.97 ± 0.83	25.33 ± 1.29	26.37 ± 2.27	3.64 ± 0.20	11.05 ± 0.2	3.06 ± 0.23	0.28 ± 0.05	59.47 ± 6.1	69.61 ± 4.5
Y25	22.79 ± 1.05	15.51 ± 1.85	18.8 ± 1.35	2.84 ± 0.16	7.48 ± 0.16	2.7 ± 0.26	0.36 ± 0.03	61.7 ± 7.5	75.17 ± 5
Y26	20.84 ± 1.36	14.95 ± 0.92	18.83 ± 1.85	2.98 ± 0.13	7.48 ± 0.13	2.60 ± 0.22	0.34 ± 0.04	56.77 ± 3.7	80.17 ± 3.4
Y27	24.55 ± 0.59	15.47 ± 1.66	19.55 ± 1.69	2.95 ± 0.15	7.71 ± 0.15	2.65 ± 0.27	0.34 ± 0.02	51.99 ± 5.1	74.99 ± 6.3
Y28	36.00 ± 0.79	28.96 ± 1.32	30.58 ± 2.05	4.24 ± 0.11	12.97 ± 0.11	3.08 ± 0.24	0.24 ± 0.05	52.77 ± 4.4	77.36 ± 4.1
Y29	25.50 ± 1.21	25.35 ± 1.15	27.32 ± 1.42	4.00 ± 0.18	11.57 ± 0.18	2.92 ± 0.21	0.25 ± 0.03	56.78 ± 6.8	75.35 ± 5.8
Y30	19.65 ± 0.93	24.14 ± 1.71	26.94 ± 1.99	4.07 ± 0.12	11.42 ± 0.12	2.84 ± 0.20	0.25 ± 0.04	69.93 ± 7.7	74.2 ± 3.6
Y31	19.87 ± 0.44	14.27 ± 1.01	18.23 ± 1.21	2.55 ± 0.19	6.90 ± 0.19	2.75 ± 0.29	0.4 ± 0.02	56.01 ± 3.4	78.72 ± 4.7
Y32	23.68 ± 1.15	15.68 ± 1.54	18.9 ± 1.71	2.71 ± 0.09	7.42 ± 0.09	2.82 ± 0.26	0.38 ± 0.05	61.24 ± 5.5	77.21 ± 6.8
Y33	23.72 ± 0.65	16.8 ± 0.87	19.81 ± 2.11	3.17 ± 0.14	8.22 ± 0.14	2.62 ± 0.23	0.32 ± 0.03	46.8 ± 4.2	76.43 ± 5.2
Y34	38.49 ± 1.31	30.02 ± 1.45	31.79 ± 1.53	4.26 ± 0.17	13.22 ± 0.17	3.19 ± 0.28	0.24 ± 0.04	46.81 ± 6.4	76.39 ± 3.3
Y35	20.98 ± 0.81	15.52 ± 1.23	19.25 ± 1.88	2.81 ± 0.10	7.54 ± 0.10	2.73 ± 0.25	0.36 ± 0.02	40.00 ± 7.2	76.51 ± 6.0
Y36	18.57 ± 1.07	14.46 ± 1.81	17.78 ± 1.39	2.94 ± 0.15	7.29 ± 0.15	2.50 ± 0.22	0.34 ± 0.05	73.56 ± 3.6	78.74 ± 4.4
Y37	14.37 ± 0.37	15.24 ± 1.18	20.09 ± 2.25	3.74 ± 0.20	8.49 ± 0.20	2.26 ± 0.27	0.26 ± 0.03	56.78 ± 5.3	76.39 ± 5.5
Y38	19.44 ± 0.91	14.41 ± 1.61	18 ± 1.61	3.11 ± 0.13	7.41 ± 0.13	2.43 ± 0.24	0.32 ± 0.04	59.69 ± 4.8	73.32 ± 3.9
Y39	18.24 ± 1.25	13.21 ± 0.99	16.84 ± 1.94	2.84 ± 0.16	6.83 ± 0.16	2.42 ± 0.21	0.36 ± 0.02	74.67 ± 6.6	76.42 ± 6.6
Y40	27.55 ± 0.54	17.61 ± 1.37	19.45 ± 1.47	2.80 ± 0.11	7.95 ± 0.11	2.87 ± 0.2	0.36 ± 0.05	70.54 ± 7.9	75.68 ± 4.0
Y41	22.34 ± 1.39	16.82 ± 1.59	19.84 ± 2.03	3.15 ± 0.18	8.18 ± 0.18	2.63 ± 0.29	0.33 ± 0.03	59.47 ± 3.3	78.78 ± 5.0
Y42	22.93 ± 0.74	16.88 ± 1.07	21.73 ± 1.79	3.28 ± 0.12	8.49 ± 0.12	2.78 ± 0.26	0.32 ± 0.04	56.78 ± 5.7	78.14 ± 3.8
Y43	21.66 ± 1.01	16.98 ± 1.76	19.5 ± 1.58	2.95 ± 0.19	7.97 ± 0.19	2.73 ± 0.23	0.34 ± 0.02	68.67 ± 4.6	73.12 ± 7.0
maximum	38.49	30.02	31.79	4.26	13.22	3.29	0.42	78.72	80.17
minimum	13.03	12.49	16.84	2.40	6.76	2.05	0.24	40	60.30
mean	23.54	17.69	20.92	3.23	8.55	2.69	0.32	59.66	75.32
SD	6.07	4.42	3.60	0.49	1.63	0.29	0.05	9.14	3.5
CV（%）	25.81	24.99	17.21	15.29	19.04	10.79	15.34	15.33	4.64

Abbreviations: 1000 SWT, thousand-seed weight; SA, seed area; SP, seed perimeter; SL/SW, seed length-to-width ratio; SL, seed length; SW, seed width; SR, seed roundness; DPPH, 2,2-diphenyl-1-picrylhydrazyl; SD, standard deviation; CV, coefficient of variation. Values are presented as mean ± standard deviation.

## Data Availability

The original contributions presented in the study are included in the article/Supplementary Materials, further inquiries can be directed to the corresponding author.

## Supplementary Materials

The following supporting information can be downloaded at https://www.mdpi.com/article/10.3390/foods15203740/s1, Figure S1: Seed morphology of 43 oat accessions; Table S1: Metabolite Identification in Digestive Products of 43 Oat Varieties.

## Abbreviations

The following abbreviations are used in this manuscript:

DPPH	2,2-Diphenyl-1-picrylhydrazyl
UPLC-MS/MS	Ultra-performance liquid chromatography–tandem mass spectrometry
OPLS-DA	Orthogonal partial least squares discriminant analysis
PCA	Principal component analysis
VIP	Variable importance in projection
MRM	Multiple reaction monitoring
