# Supplementary material for: Screening of Metabolite Biomarkers for Antioxidant and Glycosidase Inhibitory Activities from Oat Resources
Source: Foods. 2026 Oct 20;15(20):3740. doi: 10.3390/foods15203740 (PMC13704744; doi:10.3390/foods15203740)
Supplement: Supplementary file 1 [file foods-15-03740-s001.zip › Figure S1.pdf]

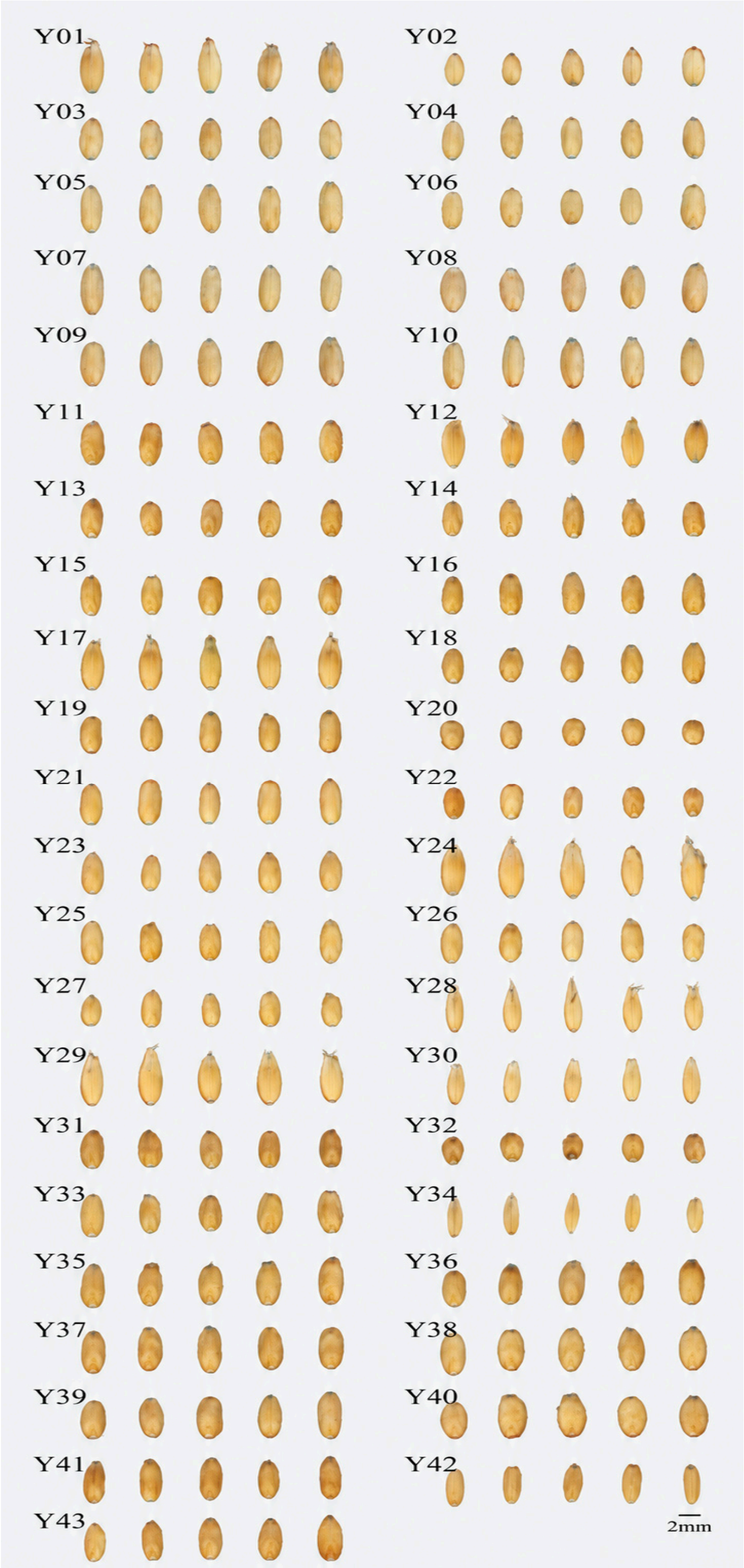

Supplementary Figure S1. Seed morphology of 43 oat accessions.
