# Supplementary material for: Screening of Metabolite Biomarkers for Antioxidant and Glycosidase Inhibitory Activities from Oat Resources
Source: Foods. 2026 Oct 20;15(20):3740. doi: 10.3390/foods15203740 (PMC13704744; doi:10.3390/foods15203740)
Supplement: Supplementary file 1 [file foods-15-03740-s001.zip › Table S1.pdf]

Table S1. Metabolite Identification in Digestive Products of 43 Oat Varieties

Library Hit	Formula	Mass (Da)	Adduct	ExtractionMass (Da)	Library Score	Intensity	Area	Classification
Trigonelline	C7H7NO2	137.14	+H	138.05496	99.4	28266	1064	Alkaloids, amines and betaines
L-Histidine	C6H9N3O2	155.15	+H	156.07675	97.3	20475	1559	Amino acids and derivatives
Ferulic acid	C10H10O4	194.18	+H	195.06519	10.3	699	129372	Phenolic acids and derivatives
Tryptamine	C10H12N2	160.22	+H	161.10732	33.3	26127	1232	Alkaloids, amines and betaines
Chlorogenic acid	C16H18O9	354.31	+H	355.10236	82.4	31,330	11167	Phenolic acids and derivatives
Glycine	C2H5NO2	75.07	+H	76.0393	91.6	31,592	50	Amino acids and derivatives
Betaine	C5H11NO2	117.15	+H	118.08626	100.0	54823	5769	Alkaloids, amines and betaines
Luteolin	C15H10O6	286.24	+H	287.05501	75.3	4,064	1410	Flavonoids and flavonoid glycosides
7-Methoxycoumarin	C10H8O3	176.17	+H	177.05462	12.5	8571	109	Coumarins
Cytosine	C4H5N3O	111.10	+H	112.05054	86.3	27,491	529	Nucleotides and their derivatives
Avenanthramide A	C16H13NO5	299.28	+H	300.08665	95.2	13,559	23	Avenanthramides
L-Phenylalanine	C9H11NO2	165.19	+H	166.08626	80.1	62,344	105490	Amino acids and derivatives

L-Leucine	C6H13NO2	131.17	+H	132.10191	99.7	359671	34348	Amino acids and derivatives
L-Glutamic acid	C5H9NO4	147.13	+H	148.06043	94.6	52383	2410	Amino acids and derivatives
L-Tyrosine	C9H11NO3	181.19	+H	182.08117	98.7	346702	19030	Amino acids and derivatives
L-Valine	C5H11NO2	117.15	+H	118.08626	93.7	85600	7431	Amino acids and derivatives
L-Arginine	C6H14N4O2	174.20	+H	175.11895	80.2	27,814	60468	Amino acids and derivatives
Leu-Ala	C9H18N2O3	202.25	+H	203.13902	40.5	13286	1480	Peptides and glutathione
N^G,N^G-Dimethyl-L-arginine	C8H18N4O2	202.26	-H	201.13570	58.7	18739	1408	Amino acids and derivatives
Myricetin 3-*O*-galactoside	C21H20O13	480.38	+H	481.09767	73.5	12,179	6746	Flavonoids and flavonoid glycosides
L-Citrulline	C6H13N3O3	175.19	+H	176.10297	16.2	4590	378	Amino acids and derivatives
benzoic acid	C7H6O2	122.12	+H	123.04406	74.4	8,001	2155	Phenolic acids and derivatives
L-Proline	C5H9NO2	115.13	+H	116.07061	100.0	17787	1737	Amino acids and derivatives
L-Isoleucine	C6H13NO2	131.17	+H	132.10191	99.3	372319	34925	Amino acids and derivatives
L-ornithine	C5H12N2O2	132.16	+H	133.09715	65	19,191	1026	Amino acids and derivatives
Reduced glutathione	C10H17N3O6S	307.32	+H	308.09108	88.2	15,125	68	Peptides and glutathione
Gly-Leu	C8H16N2O3	188.22	+H	189.12337	88.3	12567	3664	Peptides and glutathione

Nε,Nε,Nε-Trimethyl-L-lysine	C9H20N2O2	188.27	+H	189.15975	72.1	1952	154	Amino acids and derivatives
L-Aspartic acid	C4H7NO4	133.10	-H	132.03023	77.4	882	92	Amino acids and derivatives
Dihydrokaempferol	C15H12O6	288.25	+H	289.07066	69.5	13,307	166	Flavonoids and flavonoid glycosides
Isoferulic acid	C10H10O4	194.19	+H	195.06519	91.5	22,138	426	Phenolic acids and derivatives
Avenacoside B	C57H92O28	1225.34	+H	1225.58479	84	9,168	19629	Saponins and sapogenins
p-Coumaric acid O-hexoside	C15H18O8	326.30	+H	327.10744	76.2	11,648	352	Phenolic glycosides
Avenanthramide B	C17H15NO6	329.31	+H	330.09721	84.7	9,279	273	Avenanthramides
Ferulic acid O-hexoside	C16H20O9	356.33	+H	357.11801	66.5	8,732	226	Phenolic glycosides
Avenanthramide C	C16H13NO6	315.28	+H	316.08156	82.4	11,880	7865	Avenanthramides
Nuatigenin	C27H42O4	430.63	+H	431.31559	71.8	9,668	107	Saponins and sapogenins
Cycloartenol	C30H50O	426.73	+H	427.39344	87.1	10,194	239	Steroids and triterpenoids
6-Methoxycoumarin	C10H8O3	176.17	+H	177.05462	65.1	3,546	224	Coumarins
Vanillin	C8H8O3	152.15	+H	153.05462	88.2	14,249	75	Phenolic acids and derivatives
Syringaldehyde	C9H10O4	182.17	+H	183.06519	75	13,019	430	Phenolic acids and derivatives
β-Sitosterol	C29H50O	414.72	+H	415.39344	81.7	5,967	54	Steroids and triterpenoids

Sinapic acid	C11H12O5	224.21	+H	225.07575	77.5	28,224	9	Phenolic acids and derivatives
Orientin derivative	C21H20O11	448.38	+H	449.10784	81.6	6,570	9047	Flavonoids and flavonoid glycosides
L-Alanine	C3H7NO2	89.09	+H	90.05495	94.9	33,675	1512	Amino acids and derivatives
3-phenylpropionic acid	C9H10O2	150.18	+H	151.07536	54.5	5,115	1731	Phenolic acids and derivatives
Myricetin 3-*O*-glucoside	C21H20O13	480.38	+H	481.09767	85.1	11,034	296	Flavonoids and flavonoid glycosides
Protocatechualdehyde	C7H6O3	138.12	+H	139.03897	14.1	1510	63	Phenolic acids and derivatives
Syringic acid	C9H10O5	198.17	+H	199.0601	74.2	13,633	96	Phenolic acids and derivatives
p-Hydroxybenzoic acid	C7H6O3	138.12	+H	139.03897	83.8	34,358	139	Phenolic acids and derivatives
Trp-Glu	C16H19N3O5	333.34	+H	334.13975	76	15,494	329	Peptides and glutathione
Caffeic acid	C9H8O4	180.16	+H	181.04954	80.9	31,781	46528	Phenolic acids and derivatives
Uric acid	C5H4N4O3	168.11	+H	169.03562	11.1	2480	127	Nucleotides and their derivatives
α-Linolenic acid	C18H30O2	278.43	+H	279.23186	88.2	557292	28861	Lipids
Kaempferol glycoside	C21H20O11	448.38	+H	449.10784	79.6	5,014	1240	Flavonoids and flavonoid glycosides
Coumarin	C9H6O2	146.14	+H	147.04406	27.0	37309	1637	Coumarins

Protocatechuic acid	$C_7H_6O_4$	154.12	+H	155.03389	73.7	17,586	380	Phenolic acids and derivatives
Scopoletin	$C_{10}H_8O_4$	192.17	+H	193.04954	10.7	7521	535	Coumarins
fraxetin	$C_{10}H_8O_5$	208.17	+H	209.04445	51	2,721	251	Coumarins
Tricin 7-*O*-β-D-glucoside	$C_{23}H_{24}O_{12}$	492.43	+H	493.13405	84.6	10,690	14	Flavonoids and flavonoid glycosides
Glucosylvitexin	$C_{27}H_{30}O_{15}$	594.52	+H	595.16575	38.2	624	71	Flavonoids and flavonoid glycosides
6-Hydroxyflavone	$C_{15}H_{10}O_3$	238.24	+H	239.07027	100.0	3037	200	Flavonoids and flavonoid glycosides
N-acetyl-L-phenylalanine	$C_{11}H_{13}NO_3$	207.23	+H	208.09682	62.4	43,872	729	Amino acids and derivatives
Tricin	$C_{17}H_{14}O_7$	330.29	+H	331.08123	89.6	546	26	Flavonoids and flavonoid glycosides
Hypoxanthine	$C_5H_4N_4O$	136.11	+H	137.04579	85.7	20,908	717	Nucleotides and their derivatives
Guanosine	$C_{10}H_{13}N_5O_5$	283.24	+H	284.09895	100.0	4634	640	Nucleotides and their derivatives
Cytidine	$C_9H_{13}N_3O_5$	243.22	+H	244.09280	13.3	2628	288	Nucleotides and their derivatives
26-Desglucoavenacoside A	$C_{45}H_{72}O_{18}$	901.05	+H	901.47914	83.2	9,960	23591	Saponins and sapogenins
Suberosin	$C_{15}H_{16}O_3$	244.29	+H	245.11722	10.9	20708	1424	Coumarins
Rutin	$C_{27}H_{30}O_{16}$	610.52	+H	611.16066	72.5	12,676	207	Flavonoids and flavonoid glycosides

Quercetin 3-*O*-galactoside	C21H20O12	464.38	+H	465.10275	87.8	10,613	6253	Flavonoids and flavonoid glycosides
Vitexin derivative	C21H20O10	432.38	+H	433.11292	67.8	2,677	709	Flavonoids and flavonoid glycosides
